# Patient-derived organoids facilitating individual therapy in an adolescent with embryonal rhabdomyosarcoma of the cervix: a case report and literature review

**DOI:** 10.3389/fonc.2023.1241507

**Published:** 2023-09-29

**Authors:** Xinyu Qiao, Zhaomin Zeng, Peng Chen, Mingrong Xi, Minmin Hou

**Affiliations:** ^1^ Department of Obstetrics and Gynecology, West China Second University Hospital, Sichuan University, Chengdu, China; ^2^ The Key Laboratory of Birth Defects and Related Diseases of Women and Children (Sichuan University), Ministry of Education, Chengdu, China

**Keywords:** embryonal rhabdomyosarcoma of the cervix (cERMS), patient-derived organoids (PDOs), adolescent, therapy, case report

## Abstract

Rhabdomyosarcoma (RMS) is a highly aggressive pediatric neoplasm that originates from striated muscle or undifferentiated mesenchymal cells. Based on its histopathological characteristics, the World Health Organization categorizes RMS into four distinct subtypes: embryonal RMS, alveolar RMS, pleomorphic RMS, and sclerosing/spindle cell RMS. Embryonal RMS represents the predominant subtype and primarily manifests in the head and neck region, with the genitourinary system being the subsequent most frequent site of occurrence. Embryonal rhabdomyosarcoma of the cervix (cERMS) is more insidious in the reproductive tract, and there is still a lack of consensus on its treatment. Patient-derived organoids (PDOs) are being prioritized for use in guiding personalized medicine. The application of PDOs to test the sensitivity of chemotherapy drugs in patients with cERMS has rarely been reported. In this case report, we delineate the presentation and diagnosis of a 16-year-old adolescent with cERMS, emphasizing the utilization of PDOs in the management of this infrequent neoplasm. We intend to elucidate the diagnostic and therapeutic processes associated with cERMS by referencing previously reported literature on this infrequent tumor, aiming to offer a foundation for clinical practice.

## 1 Introduction

Rhabdomyosarcoma (RMS) is a malignant neoplasm of mesenchymal derivation and constitutes the predominant soft tissue sarcoma in pediatric and adolescent populations (1). Based on histological classification, the embryonal subtype is predominant (2). The head, neck, and genitourinary tract represent the most common sites of occurrence for embryonal RMS (3). Within the female reproductive tract, this neoplasm is more frequently encountered in the vagina and is rarer in the cervix. Embryonal rhabdomyosarcoma of the cervix (cERMS) predominantly affects young women aged between 12 and 26 years (3).

The predominant clinical manifestation is vaginal bleeding, accompanied by soft, polypoid, grape-like masses. Pathologically, cERMS is characterized by the cambium layer, which is denoted by a subepithelial accumulation of tumor cells (4). A significant proportion of patients with cERMS possess an inherited DICER1 mutation, which predisposes individuals to a spectrum of tumor syndromes (5). In almost 50% of the cases, foci of cartilage are discerned, a feature that appears distinctively associated with embryonal RMS in the context of DICER1 mutations (5). Immunohistochemically, the neoplastic cells are positive for Desmin, MyoD1, and Myogenin (4).

A unified treatment strategy for this uncommon tumor type remains elusive. With a multimodal treatment approach, including surgical intervention, and systemic chemotherapy with considerations for radiotherapy, the survival for RMS patients has been greatly improved (6). The overall survival rate approaches 80% with appropriate treatment. cERMS demonstrates chemosensitivity to combination therapy, notably with vincristine, actinomycin D, and cyclophosphamide (VAC) (3). Nonetheless, considerable heterogeneity is observed across individual patients.

Three-dimensional (3D) organoid culture of human tumor tissue has emerged as a representative platform to model tumor genetic and phenotypic features *in vitro* (7). As such, patient-derived organoids (PDOs) are increasingly recognized for their potential to steer personalized medicine. To date, there is an absence of literature detailing the utilization of PDOs for assessing chemotherapy drug sensitivities in cERMS patients. In this case study, we discuss the presentation of a 16-year-old adolescent diagnosed with cERMS. We elucidate the potential utility of PDOs in refining therapeutic decisions for cERMS, and draw upon extant literature on this rare pathology to offer insights for clinical practice.

## 2 Case presentation

A 16-year-old adolescent presented with a history of “irregular vaginal bleeding lasting over a year, which had worsened over the past six months.” She was subsequently referred to the gynecology department of our institution on November 17, 2022. In October 2021, she experienced an episode of unprovoked, painless vaginal bleeding, which was overlooked and thus remained untreated. On November 14, 2022, due to symptoms of dizziness, fatigue, and palpitations, she sought care at a local hospital. Laboratory evaluation revealed severe anemia with a hemoglobin level of 34g/L. Magnetic resonance imaging (MRI) of the pelvis disclosed a substantial vaginal mass, which extended anteriorly into the anterior fornix and posteriorly into the uterorectal pouch. The local hospital administered a blood transfusion, comprising 7.0 U of leuko-depleted suspended red blood cells and 300ml of frozen plasma, along with hemostatic and other symptomatic interventions. The preliminary diagnosis leaned heavily towards a vaginal or cervical malignancy. Once her condition stabilized, she was referred to our center for further evaluation and management.

Upon physical examination, the patient exhibited signs of anemia and had a Grade II bilateral thyroid enlargement. A rectal examination revealed a cystic-solid mass, approximately 10 cm in size, located anterior to the rectum. The mass had well-defined margins, and the deeper pelvic structures could not be palpated through the examination.

Laboratory findings indicated anemia, with a hemoglobin level of 92g/L. Tests for tumor markers, thyroid function, coagulation profile, and other relevant indicators yielded results within the normal range, with no significant abnormalities detected.

**Figure 1:**
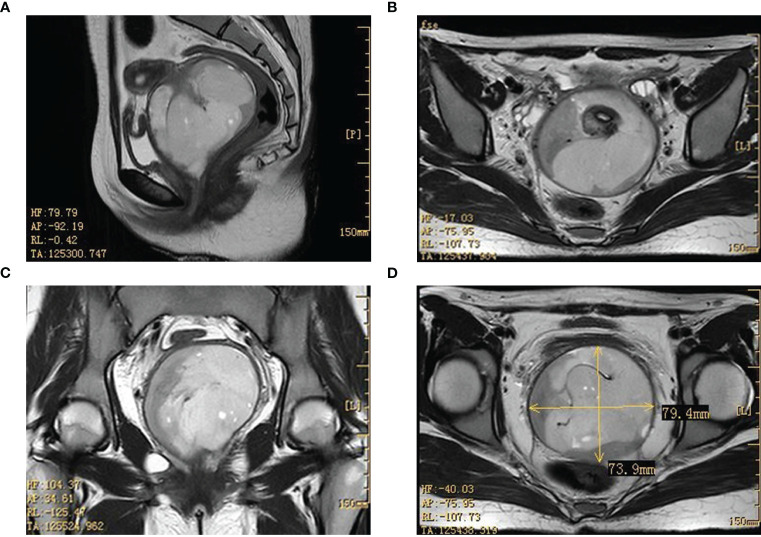
Imaging results in the pelvic lesion tissue. The results of the MRI showed a mixed signal mass in the external cervical orifice and vaginal fornix (size about 7.4×7.9×8.5cm). (**A**, **C**: sagittal position; **B**, **D**: horizontal position). MRI, magnetic resonance imaging.

To gain a deeper understanding of the patient’s condition, imaging studies were conducted. Ultrasound imaging revealed a hypoechoic mass, measuring approximately 8.6×6.1×8.0 cm, situated in the vaginal region just below the uterus. A pelvic MRI further identified a mass with mixed signal intensity in the region of the external cervical and vaginal fornix, measuring about 7.4×7.9×8.5 cm. This mass extended upward, infiltrating the mid and lower cervical muscle layers, occupied the vaginal fornix, and extended downward into the mid and upper segments of the vagina. The MRI findings strongly indicated a malignant process (**Figure 1**). The corpus of the uterus, both adnexal, and bilateral obturator lymph nodes, and the walls of the bladder and rectum displayed no abnormalities.

**Figure 2:**
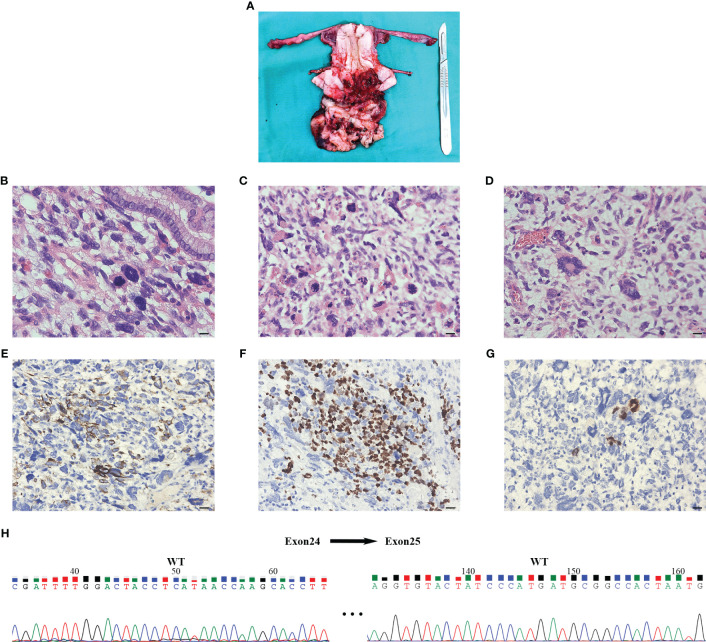
The results of postoperative pathology and Sanger sequencing. **(A)** An ulcerated mass was seen at the external cervical orifice (size about 7cm x 8cm x 7cm) and presented as a tongue-shaped excrescence into the vagina. The cut surface was grayish solid with extensive hemorrhage and necrosis, invading the superficial layer of the cervical stroma. **(B-D)** Postoperative pathology of hematoxylin-eosin staining indicated that the tumor was a cERMS: Small, spindled, and round cells were located in a background of myxoid stroma suggestive of rhabdomyoblastic differentiation. The multinucleated giant cells could be seen in the lesion and nuclear fission is obvious (×400, Scale bar=20mm). **(E-G)** Immunochemical staining showed that the tissue was Desmin, MyoD1, and Myogenin positive (×400, Scale bar=20mm). **(H)** Sanger sequencing of tumor tissue did not detect DICER1 gene mutation in exons 24 and 25. The numerical annotations were the actual nucleotide order on the exons 24 and 25. cERMS, embryonal rhabdomyosarcoma of the cervix.

Vaginal endoscopic and histologic evaluation of the tumor was used to confirm tumor malignancy, tissue origin, and classification (**Supplementary video**). Intraoperative rapid cytopathology was suspected to be a cERMS. Guided by the Chinese recommendations for the diagnosis and treatment of rhabdomyosarcoma in pediatric and adolescent populations, as well as the NCCN clinical practice guidelines in oncology, the patient underwent an abdominal radical hysterectomy, bilateral salpingectomy, bilateral pelvic lymphadenectomy, and paraaortic lymph node sampling (8, 9). The excised uterus and associated tumor specimens are presented in **Figure 2A**. Pathological evaluation and subsequent immunohistochemical (IHC) staining confirmed the diagnosis of cERMS (**Figures 2B-G**). Hematoxylin-eosin (HE) staining showcased the presence of mitotic figures and giant tumor cells. Immunohistochemical staining discovered positivity for Desmin, MyoD1, and Myogenin. The Ki-67 proliferation index was approximately 65%. Concurrently, genetic analysis was conducted to corroborate the clinical diagnosis. To evaluate the potential presence of DICER1 mutations, hotspot regions of exons 24 and 25, which code for the RNase III b domain, were amplified using PCR and subsequently sequenced using the Sanger method from the patient’s tumor tissue. However, no DICER1 gene mutations were detected in these exons (**Figure 2H**).

Based on the staging criteria set forth by the Intergroup Rhabdomyosarcoma Study Group (IRSG), the patient was categorized as being in stage Ia. For subsequent postoperative management, the VAC combination chemotherapy regimen was contemplated for the patient, given its status as the contemporary first-line therapeutic approach for cERMS (8).

**Figure 3:**
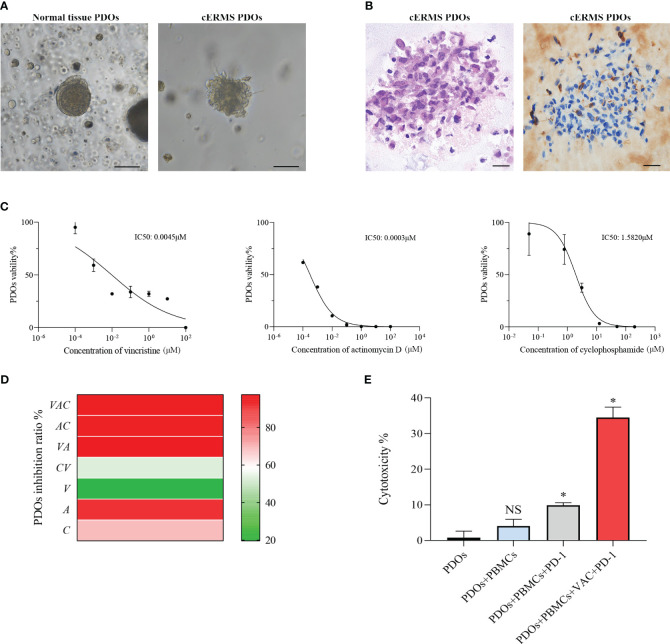
The drug sensitivity test of PDOs. **(A)** Representative bright field images of normal tissue PDOs and cERMS PDOs prior to drug sensitivities (Scale bar=100μm). **(B)** Representative hematoxylin-eosin staining and immunochemical staining of Desmin images of cERMS PDOs prior to drug sensitivities (Scale bar=20μm). **(C)** Cell viability of PDOs treated with vincristine, actinomycin D, and cyclophosphamide. PDOs viability (%)=(OD Sample-OD Blank)/(OD Control-OD Blank)×100%. **(D)** The inhibition ratio of PDOs treated with the single or combination of vincristine, actinomycin D, or cyclophosphamide. PDOs Inhibition Ratio (%) = (1-(OD Sample-OD Blank)/(OD Control-OD Blank))×100%. **(E)** The cytotoxicity of the co-culture model of PBMCs and cERMS PDOs treated with the single PD-1 or combined with VAC. PDOs, patient-derived organoids; V, vincristine; A, actinomycin D; C, cyclophosphamide; PD-1, Programmed death protein 1; PBMCs, peripheral blood mononuclear cells; NS, no statistical significance; *P<0.05.

After written informed consent, we generated organoids from the patient’s resected cERMS tissue to further clarify the chemotherapeutic drug sensitivity. The PDOs were produced using tumor and surrounding normal samples based on established methods, with adaptations (1, 10). Briefly, the patient samples were washed with DMEM/F12 medium (12634010, Thermo), minced into small pieces, digested with collagenase type II (1mg/ml, C2-28-100MG, Sigma) for 1 h in a 37 °C water bath, and passed through a 70μm filter. The cell pellet was then resuspended in the Matrigel matrix (356255, Corning) while on ice. Following this, it was transferred to a culture plate and allowed to incubate in a 37°C environment with 5% CO_2_ for a duration of 30 minutes. Upon the Matrigel matrix completed solidification, an expansion medium was added. The PDOs were then placed in a 37°C carbon dioxide cell incubator for cultivation. Subsequently, the PDOs were passaged according to their growth patterns. Before drug sensitivity testing, the PDOs exhibited solid spheroid morphology with irregular edges (**Figure 3A**). HE and IHC staining revealed that the cultured PDOs preserved key phenotypic features of the parent cERMS, including nuclear pleomorphism, mitotic rate, and immunoreactive profiles (**Figure 3B**).

The chemosensitivity of cERMS PDOs against standard chemotherapy agents including vincristine, actinomycin D, and cyclophosphamide was assessed. To compare the drug sensitivities of the tested drugs, the half-inhibitory concentrations (IC50) of each drug were determined using “GraphPad Prism 8.0” (**Figure 3C**). Among them, actinomycin D exhibited the most potent tumor growth inhibition, with an IC50 of 0.0003μM. This was followed by vincristine and cyclophosphamide, which had IC50 values of 0.0045μM and 1.5820μM, respectively. The VAC combination therapy demonstrated a tumor inhibition rate of 95.87%, which was superior to monotherapy or the combination of any two drugs (**Figure 3D**). Additionally, 2 mL of peripheral blood was collected from the patient for the isolation of peripheral blood mononuclear cells (PBMCs) using Ficoll-Paque density-gradient separation. After lysing the red blood cells, the obtained PBMCs were cultured in RPMI 1640 medium, supplemented with 10% Fetal Bovine Serum, 1% penicillin/streptomycin, and 25 ng/mL of recombinant human IL-2 (200-0, Thermo). Subsequently, the PBMCs were quantified and co-cultured with PDOs at a ratio of 50–100:1. In the co-culture model of PBMCs and cERMS PDOs, VAC and immune checkpoint inhibitors have synergistic anti-tumor effects. The results showed that the combined use of the Programmed death protein 1 (PD-1) inhibitor and VAC achieved a favorable therapeutic effect, with cytotoxicity reaching 34.49% (**Figure 3E**). Given the risk of non-specific immune activation by the PD-1 inhibitor, which could lead to immune-mediated injury in other organs, we have opted to position the PD-1 inhibitor as a second-line therapeutic strategy.

**Figure 4:**
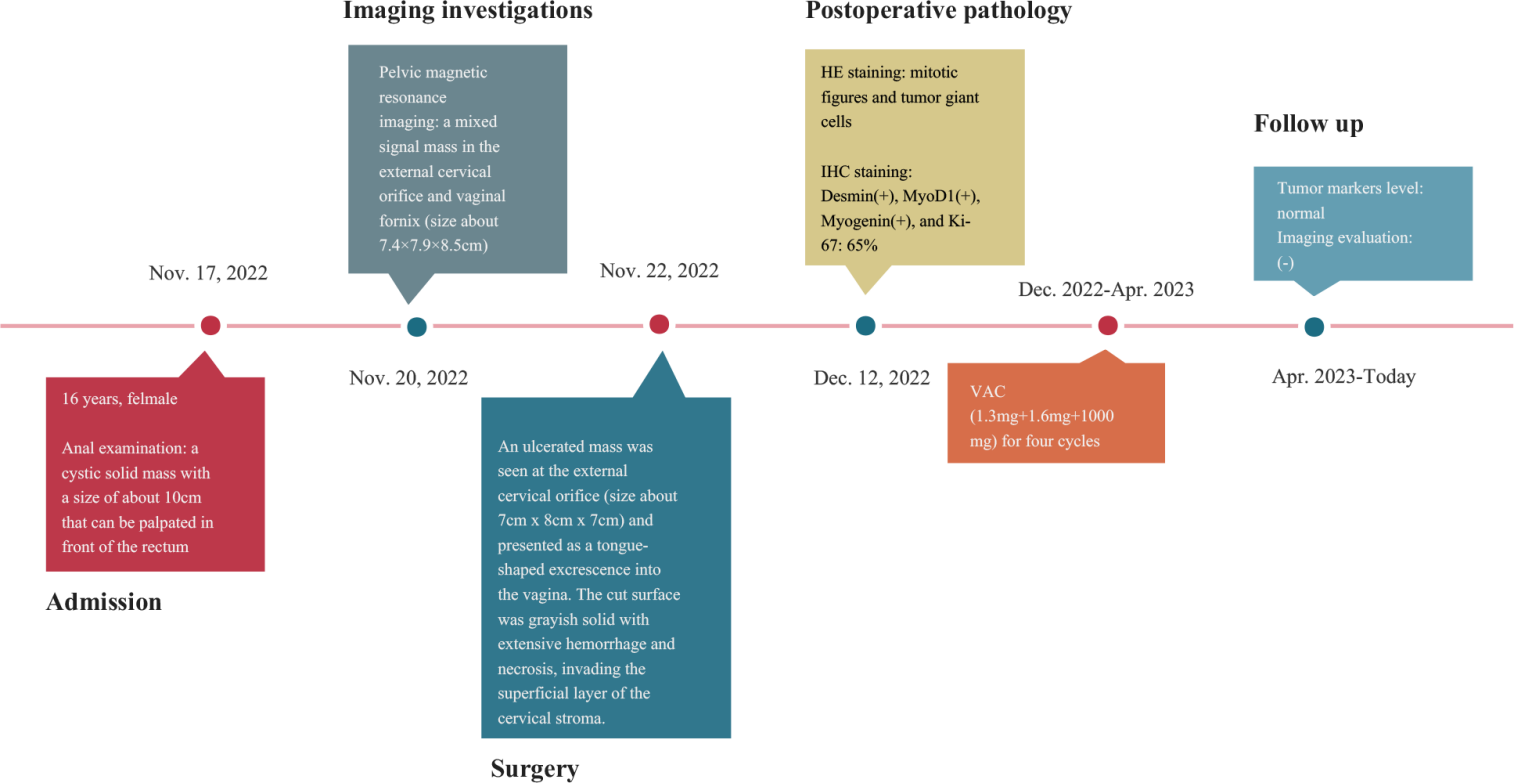
The diagnosis and treatment strategy timeline schematic.

According to the recommendation and drug sensitivity results, the patient has been treated with VAC chemotherapy(1.3mg+1.6mg+1000mg) for four cycles. During periodic follow-up evaluations, recurrence was not observed, the tumor markers level remained normal, and MRI did not show any new lesion. The diagnosis and treatment strategy timeline schematic are presented in **Figure 4**.

## 3 Discussion

We report a detailed clinical case of a 16-year-old adolescent, with cERMS and multinodular goiter. A comprehensive histopathological characterization and a Sanger sequencing analysis of this rare tumor are provided. In addition, PDOs were used to reflect drug sensitivities already known for cERMS and to be of use for such screening approaches.

RMS represents the predominant soft tissue sarcoma observed in children and adolescents, comprising 2-3% of all malignancies diagnosed within this demographic (11). According to the latest World Health Organization pathological classification, RMS is categorized into four subtypes: embryonal, alveolar, spindle cell/sclerosing, and pleomorphic (12). Embryonal RMS is the predominant subtype, with the highest incidence in the head and neck areas, succeeded by its occurrence in the genitourinary system (2). Within the female reproductive system, the vagina is the primary site affected by embryonal RMS, while a mere 0.5% or fewer cases manifest in the cervix (13). Embryonal RMS accounts for 0.4%-1.0% of all the malignant tumors of the cervix, and it is usually seen in the second decade of life (2).

cERMS typically presents with vaginal bleeding, protruding vaginal mass, cervical polyp, or expelled tumor fragments through the vaginal (4). Our case manifested with pronounced anemia resulting from vaginal bleeding, accompanied by a substantial mass in the vagina. In the literature, other reported cases have exhibited symptoms such as abdominal pain, dyspareunia, and post-coital bleeding (2, 3). It’s imperative for obstetricians and gynecologists to be vigilant regarding these atypical clinical presentations, particularly in young adults.

The majority of cERMS typically present as a polypoid or grape-cluster-like appearance, being soft in texture, and they might be associated with hemorrhage and necrosis (4). However, many cERMS grossly resemble simple polyps and correct diagnosis is obtained only at the time of recurrence (14). Microscopically, cERMS is characterized by several distinct pathological features, including the presence of cambium layers, areas of rhabdomyoblastic differentiation, zones of anaplasia, and regions showcasing chondroid differentiation (15, 16). The cambium layer is constituted by a subepithelial accumulation of primitive blue spindle or ovoid cells. The hallmark of rhabdomyoblastic differentiation is the presence of copious eosinophilic cytoplasm and/or the manifestation of positive rhabdoid markers, with diffuse Desmin positivity and potential focal expression of MyoD1 and Myogenin (16). Additionally, smooth muscle actin (SMA) and hormonal receptors are usually negative in cERMS (15, 16). Among these histopathologic features, foci of chondroid differentiation are a positive prognostic factor, while deep myometrial invasion, lymphatic invasion, and a focal alveolar pattern are correlated with an adverse prognosis (3).

Previous studies have shown that nearly all cERMS harbor a DICER1 mutation (11, 16, 17). The DICER1 locus is situated on chromosome 14q32.13 and contains 27 exons. DICER1 belongs to the ribonuclease III (RNase III) family and is pivotal in both the RNA interference pathway and the canonical miRNA biogenesis pathway (18). During the course of tumorigenesis, a germline mutation of one DICER1 allele is not sufficient to cause tumorigenesis until a second somatic mutation of the other allele, which is usually located in the RNase III b domain (19). These mutations may cause activation of oncogenes through dysregulation of miRNA, and then contribute to tumorigenesis of multiple systems (20). Besides cERMS, tumors associated with the DICER1 include pleuropulmonary blastoma, multinodular goiter, cystic nephroma, Sertoli–Leydig cell tumor of the ovary, and other rare tumor entities (5).

Since our patient had both cERMS and multinodular goiter, DICER1 tumor predisposition syndrome was highly suspected. Hotspot mutations of DICER1 were examined in the patient’s tumor sample through PCR amplification of exons 24 and 25 (the DNA sequences encoding the RNase III b domain) and subsequently analyzed via Sanger sequencing (16). However, we did not detect mutations in the DICER1 gene (exons 24 and 25). This outcome could be attributed to the limited sensitivity of Sanger sequencing and the incomplete coverage of the coding exons for the RNase III b domain (21). Owing to the substantial time and financial costs, we did not perform next-generation sequencing. It could not be ruled out that the combination of cERMS with multinodular goiter, in this case, is a coincidence. This supposition is further bolstered by the tumor tissue’s lack of cartilage differentiation, given that foci of cartilage are a distinctive characteristic of embryonal RMS associated with DICER1 mutations (5, 16).

Given the rarity of cERMS, standardized guidelines are absent, compelling physicians to rely on experiential insights from published case reports and series. In clinical practice, the primary treatment approach for cERMS continues to be a combination of surgery, chemotherapy, and/or radiotherapy. At present, the methods of surgical treatment mainly include radical hysterectomy with or without lymphadenectomy, vaginectomy, cervicectomy, and local lesion excisions. Since cERMS tend to occur in young women, fertility-sparing is a major concern. The IRSG states fertility-sparing surgery and chemotherapy appropriate treatments for patients with localized disease but not applicable for advanced-stage and metastatic disease (2). Some case reports have been published that show the effectiveness and security of fertility-sparing treatment (13, 22). However, random controlled trials with high quality are needed to compare the prognosis of radical hysterectomy and fertility-sparing surgery for cERMS.

According to the recommendations of our country and the NCCN clinical practice guidelines in oncology, abdominal radical hysterectomy, bilateral salpingectomy, bilateral pelvic lymphadenectomy, and paraaortic lymph node sampling were performed for this patient (8, 9). The extent of postoperative residual disease is an important prognostic factor (13). The IRSG divided the status after the primary surgery into 3 groups and analyzed the survival (13, 23). The patients with no residual tumor after surgery (Clinical Group I) had a 5 years survival rate of approximately 90%, but the patients with macroscopic residual disease (Clinical Group III) had a 5 years survival rate of less than 70% (23). According to the IRSG postsurgical staging, our patient was placed in Clinical Group I and had no residual disease after surgery (8).

It is generally recognized that adjuvant chemotherapy is essential. Montag et al. in 1986 reported a patient who underwent adequate surgical excision and chemotherapy survived for 7 years without recurrence (24). Subsequent randomized studies also have shown that the addition of adjuvant chemotherapy makes a substantial contribution to the improvement in survival (25). Currently, combination therapy with VAC is the standard chemotherapeutic regime for RMS (8). However, different patients with the same tumors often present diverse phenotypes that dynamically evolve throughout disease progression and clinical treatment (7).

Organoids as *in vitro* 3D culture systems of human tumor tissue can model histopathological, genetic, and phenotypic heterogeneity across different patients (26). PDOs can implement individualized therapy by providing a mechanism for reliably testing drug sensitivity and IC50 value (27). In particular, for many cancer subtypes of epithelial origin, PDOs can be a predictive biomarker for treatment response (27, 28). The feasibility of using PDOs on nonepithelial tumors has only recently been shown. In 2022, Meister et al. made the first demonstration that PDOs are also applicable to tumors of entirely mesenchymal origin (such as rhabdomyosarcoma), and the models can be used for both drug screening and genetic editing (1). Accordingly, we generated organoids from the patient’s resected cERMS tissue.

As the first chemotherapy option for this patient, VAC was considered. To identify potentially effective drugs, we used the PDOs for *in vitro* drug response testing. Vincristine and actinomycin D indeed showed inhibition for cERMS organoids growth, with IC50 of 0.0045 and 0.0003μM respectively. Furthermore, the effect of VAC combination therapy (inhibition rate: 95.87%) was better than that of monotherapy. With an IC50 of 1.5820μM, cyclophosphamide demonstrated a meager antitumoral impact. This is in line with previous studies showing that cyclophosphamide did not contribute to the success of the treatment, but significantly reduced treatment toxicity (22). Furthermore, PD-1 inhibitor could induce immune cells to kill tumor cells *in vitro* co-culture model of PBMCs and cERMS PDOs. There is clinical evidence that a patient with refractory RMS was controlled by PD-1 inhibitor with a recurrence-free survival lasting more than 3 years (29). However, non-specific activation of the immune system caused by PD-1 inhibitors may lead to immune damage to other organs and tissues. It becomes important to discuss the PD-1 inhibitor as a posterior-line treatment for cERMS.

In conclusion, cERMS is a rare malignancy in the uterine cervix. When a patient has vaginal bleeding in addition to a large cervical polyp, it should be considered. The diagnosis of cERMS is greatly aided by proper immunohistochemistry in conjunction with histopathology. The prognosis of patients with cERMS can be significantly increased by early detection and treatment. Surgery combined with chemotherapy and/or radiotherapy is the main treatment for cERMS. This study describes the first reported use of cERMS organoids to identify sensitivity to chemotherapy drugs and facilitate individual-based treatment. In the co-culture model of PBMCs and cERMS PDOs, a synergistic antitumor effect was observed with the combination of VAC and the PD-1 inhibitor. This suggests the potential for improved patient outcomes. However, further validation is essential.

## Data availability statement

The original contributions presented in the study are included in the article/**Supplementary Material**. Further inquiries can be directed to the corresponding author.

## Ethics statement

The studies involving human participants were reviewed and approved by the Ethics Committee of West China Second University Hospital. The studies were conducted in accordance with the local legislation and institutional requirements. Written informed consent for participation in this study was provided by the participants’ legal guardians/next of kin. Written informed consent was obtained from the individual(s), and minor(s)’ legal guardian/next of kin, for the publication of any potentially identifiable images or data included in this article.

## Author contributions

XQ and ZZ composed the manuscript and literature review. MX and PC guided the analysis of radiological images and histological images analysis and enriched the discussion. MH reviewed the revised paper and provided the final approval of the version to be published. All authors contributed to the article and approved the submitted version.
